# *TP53* and *MDM2* single nucleotide polymorphisms influence survival in non-del(5q) myelodysplastic syndromes

**DOI:** 10.18632/oncotarget.5255

**Published:** 2015-09-25

**Authors:** Kathy L. McGraw, Thomas Cluzeau, David A. Sallman, Ashley A. Basiorka, Brittany A. Irvine, Ling Zhang, P.K. Epling-Burnette, Dana E. Rollison, Mar Mallo, Lubomir Sokol, Francesc Solé, Jaroslaw Maciejewski, Alan F. List

**Affiliations:** ^1^ Department of Malignant Hematology, Moffitt Cancer Center and Research Institute, Tampa, FL, USA; ^2^ Hematology Department, CHU of Nice, Nice, France; ^3^ University Nice Sophia Antipolis, Faculty of Medicine, Nice, France; ^4^ Mediterranean Center of Molecular Medicine, INSERM U1065, Nice, France; ^5^ French Group of Myelodysplasia, France; ^6^ Moffitt Cancer Center and Research Institute and The Cancer Biology Ph.D. Program, University of South Florida, Tampa, FL, USA; ^7^ Department of Pathology, Moffitt Cancer Center and Research Institute, Tampa, FL, USA; ^8^ Department of Immunology, Moffitt Cancer Center and Research Institute, Tampa, FL, USA; ^9^ Department of Cancer Epidemiology, Moffitt Cancer Center and Research Institute, Tampa, FL, USA; ^10^ Institut de Recerca Contra la Leucèmia Josep Carreras (IJC) Badalona, Barcelona, Spain; ^11^ Cleveland Clinic, Taussig Cancer Institute, Cleveland, OH, USA

**Keywords:** TP53, MDM2, myelodysplastic syndromes, single nucleotide polymorphisms, survival

## Abstract

P53 is a key regulator of many cellular processes and is negatively regulated by the human homolog of murine double minute-2 (MDM2) E3 ubiquitin ligase. Single nucleotide polymorphisms (SNPs) of either gene alone, and in combination, are linked to cancer susceptibility, disease progression, and therapy response. We analyzed the interaction of *TP53* R72P and *MDM2* SNP309 SNPs in relationship to outcome in patients with myelodysplastic syndromes (MDS). Sanger sequencing was performed on DNA isolated from 208 MDS cases. Utilizing a novel functional SNP scoring system ranging from +2 to −2 based on predicted p53 activity, we found statistically significant differences in overall survival (OS) (*p* = 0.02) and progression-free survival (PFS) (*p* = 0.02) in non-del(5q) MDS patients with low functional scores. In univariate analysis, only IPSS and the functional SNP score predicted OS and PFS in non-del(5q) patients. In multivariate analysis, the functional SNP score was independent of IPSS for OS and PFS. These data underscore the importance of *TP53* R72P and *MDM2* SNP309 SNPs in MDS, and provide a novel scoring system independent of IPSS that is predictive for disease outcome.

## INTRODUCTION

The myelodysplastic syndromes (MDS) share phenotypic features of dysplastic and ineffective hematopoiesis accompanied by remarkable hematologic, genetic and clinical heterogeneity. Although clinical scoring systems provide tools for prognostic discrimination, they overlook biologic features potentially relevant to disease behavior [1]. The tumor suppressor gene, *TP53*, is a key regulator of many cellular processes, and is a key driver of hematologic features of disease in MDS. P53 is particularly important in patients with MDS harboring an interstitial deletion of chromosome 5q [del(5q)], where *RPS14* haploinsufficiency results in ribosomal stress liberating free ribosomal proteins that bind to, and trigger degradation of the primary negative regulator of p53, the human homologue of murine double minute-2 (MDM2) [2–4]. *TP53* mutations are found in the vast majority of solid tumors [5]. However, in MDS, *TP53* mutations are demonstrable in approximately 10% of all cases, 20% of del(5q) cases, and more than 70% of cases with a complex karyotype [6, 7]. Mutations involving the DNA binding domain (DBD) of *TP53* carry a particularly poor prognosis [7]. Upregulation of MDM2 has also been observed in many malignancies including up to 10% of MDS cases [8]. Recently, we demonstrated the importance of MDM2 in the activity of lenalidomide, whereby inhibition of the E3 ubiquitin ligase activity of MDM2 resulted in stabilization of the protein and corresponding degradation of p53 in del(5q) MDS, illustrating the critical role of these proteins in MDS disease biology, progression and therapeutic response [4].

Single nucleotide polymorphisms (SNPs) of both *TP53* and *MDM2* have been linked to earlier cancer onset, greater cancer risk, and response to therapy [9–13]. The non-synonymous, SNP of *TP53*, R72P at codon 72, resides in exon 4 outside the DBD, and has been linked to cancer susceptibility in patients with solid tumors [14, 15]. Among hematologic malignancies, associations between the R72P SNP and clinical features have been conflicting [9, 10, 16, 17]. A G- to C-allele substitution results in an arginine to proline amino acid change, predictably affecting the structure of the SH-3 domain, and the functionality of the protein [18, 19]. For example, the C-allele has been shown to have diminished apoptotic potential *in vitro* [19]. In a recent study of more than 700 MDS patients, we found no overall association of R72P alone with disease outcome in MDS; however, there was a trend towards inferior survival with the G-allele in patients with del(5q) MDS, and a significant association of this allele with telomeric deletions involving 5q34 [17]. In non-del(5q) MDS, C-allele homozygozity was associated with non-significantly inferior survival demonstrating the differential impact of the p53 SNP in cytogenetically distinct MDS populations [17].

A well-studied SNP in *MDM2*, SNP309, has also been linked to increased cancer risk [12, 13]. Here, substitution of the ancestral T-allele with a G-allele introduces an additional Sp1 transcription factor binding site in the *MDM2* promoter. This leads to increased MDM2 expression, and decreases in the cellular levels of p53. In hematological malignancies, the *MDM2* SNP309 G-allele is associated with increased risk for acute myeloid leukemia (AML) and chronic myelogenous leukemia (CML) [20, 21]. Additionally, there have been a number of studies analyzing the effects of R72P and SNP309 interactions in solid tumors, demonstrating combined effects on clinical features and prognosis of disease [22–24]. Previous reports of these SNP combinations in MDS did not distinguish between del(5q) and non-del(5q) MDS patients [16].

In this study, we examine the effect of the combination of *TP53* R72P and *MDM2* SNP309 on clinical features of del(5q) and non-del(5q) MDS, and find significant differences in survival based on genotypic interaction.

## RESULTS

### Patient demographics

We analyzed 208 MDS patients [95 non-del(5q), 102 del(5q), and 11 with unknown cytogenetics]. The median OS of our cohort was 52.9 months [40.3–65.7]. Median age at diagnosis was 71 [range 27–89]. The male to female ratio was 111/97. The distribution of IPSS category, cytogenetic risk group, WHO subclassification, and genotype frequencies are summarized in Table 1. Patient cytogenetics are listed in Supplemental Table 1.

**Table 1:** Patient demographics

	*n* = 208
**Median age (range)**	71 (27–89)
**Sex ratio M/F**	111/97
**Race** Caucasian Non Caucasian	189 (91%) 19 (9%)
**WHO classification (%)** MDS with Isolated 5q Other^1^ RAEB-1 RAEB-2	20 (10%) 83 (35%) 30 (14%) 11 (5%)
**IPSS cytogenetic risk** Good Intermediate Poor	137 (66%) 24 (12%) 31 (15%)
**IPSS** Low Intermediate-1 Intermediate-2 High	105 (50%) 72 (35%) 25 (12%) 4 (2%)
**IPSS-R** Very Low Low Intermediate High Very High	85 (51%) 20 (35%) 47 (12%) 14 (7%) 15 (7%)

*n* (%)

1 Other (RA, RARS, RCMD, RCMD-RS)

Missing data was excluded from table

### Functional SNP scoring system predicts outcome

**Figure 1:**
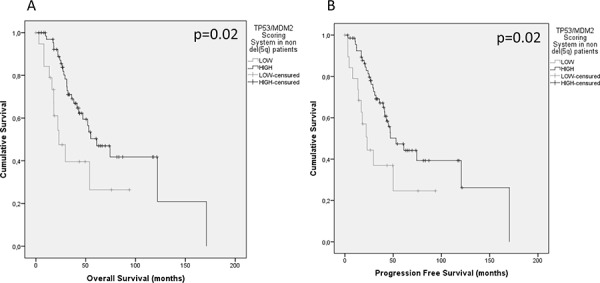
Kaplan-Meier plots of survival based upon SNP functional score **A.** OS and **B.** PFS of low scoring (score of less than 0) and high scoring patient cohorts (score equal to or greater than 0) based on weighted R72P and SNP309 p53 activity score.

In a recent study, we found there was no significant association of R72P genotype alone with survival in either del(5q) or non-del(5q) MDS [17]. Here, analysis of the *MDM2* SNP309 genotype alone also demonstrated no influence on either OS [*p* = 0.419, non-del(5q); *p* = 0.123, del(5q)] or PFS [*p* = 0.193, non-del(5q); *p* = 0.612, del(5q)]. In order to analyze the interactions of R72P and SNP309 encoded proteins, we created a SNP functional score based upon predicted p53 activity. As the G-allele in SNP309 increases MDM2 expression, thereby enhancing p53 degradation, and the R72P C-allele has diminished apoptotic potential [19], we weighted the MDM2/TP53 GG/CC genotype combination lowest with a score of − 2. Conversely, the TT/GG genotype combination had the greatest score at +2. The double heterozygotes were assigned a score of 0, and all intermediate genotype combinations are summarized in Table 2. Patients were then stratified into either high p53 functional score (equal to or greater than 0) or low p53 functional scoring groups (below 0). We did not discern any significant associations with age, sex, race, WHO subclassification, cytogenetic risk group, IPSS, or IPSS-R in either del(5q) or non-del(5q) MDS, or within the entire patient cohort. These analyses and their corresponding *p*-values are summarized in Table 3. We then applied the functional SNP scoring system to assess relationship to OS and found there was no significant association among the combined MDS cases (*p* = 0.54) with similar results for PFS (*p* = 0.66). Median OS was 53.9 months (19.6–88.3) and 54.0 months (40.2–67.8) in low and high scorers, respectively. Median PFS was 50.0 months (20.2–79.8) and 46.9 months (36.8–57.0) in low and high scorers, respectively. Given the importance of p53 in the physiopathology of del(5q) MDS as a result of ribosomal stress, the greater incidence of *TP53* mutations in this MDS subtype, as well as our previous study of R72P SNP alone, it was imperative that we analyze del(5q) MDS and non-del(5q) MDS separately. We found a statistically significant difference in OS based on the functional SNP score in non-del(5q) MDS patients (*p* = 0.02). Median OS in high scorers was 61.0 months (37.7–84.4) compared to a median OS of 23.0 months (9.9–36.1) in low scorers (Figure 1). We then analyzed PFS and found a statistically significant increase in PFS in those patients with a high functional SNP score compared to those with the lower SNP score [*p* = 0.02, median PFS was 53.0 months (33.0–73.0) for high and 23.0 months (13.7–32.3) for low] (Figure 1). In del(5q) MDS patients, we did not observe significant differences in OS or PFS according to functional SNP score. Median OS was 77.9 months (45.4–110.5) and 47.5 months (29.0–66.1) in low and high score groups, respectively (*p* = 0.27), while median PFS was 77.9 months (45.4–110.5) vs. 41.9 months (28.0–66.1) in low and high score cohorts, respectively (*p* = 0.19).

**Table 2:** *TP53/MDM2* scoring system

*MDM2* SNP309/*TP53* R72P	Points
GG/CC	− 2
TT/CC	− 1
GG/GC or TG/CC	− 0.5
TG/CG	0
TT/GC or TG/GG	0.5
GG/GG	1
TT/GG	2
	
**Sub-groups**	**Points**
Score Low	<0
Score High	≥0

**Table 3:** Correlations between clinical parameters and p53 functional SNP scoring system

	Non-del(5q) MDS		Del(5q) MDS patients	
Functional SNP Scoring System	Low *n* = 20	High *n* = 75	Low *n* = 26	High *n* = 76
**Median age** (range)	70 (38–85)	71 (27–89) *p = 0.89*	69 (45–82)	72 (27–89) *p = 0.90*
**Sex ratio M/F**	15/5	52/23 *p = 0.62*	7/19	32/44 *p = 0.17*
**Race** Caucasian Non Caucasian	20(100%) 0	68 (91%) 8(9%) *p = 0.92*	24 (92%) 2 (8%)	68 (86%) 8 (14%) *p = 0.87*
**WHO classification** MDS with Isolated 5q Other^1^ RAEB-1 RAEB-2	0 13 (65%) 4 (20%) 3 (15%)	0 64 (85%) 6 (8%) 3 (4%) *p = 0.06*	9 (35%) 10 (38%) 4 (15%) 2 (8%)	25 (32%) 29 (36%) 15 (18%) 6 (8%) *p = 0.95*
**IPSS cytogenetic risks** Good Intermediate Poor	17 (85%) 1 (5%) 2 (10%)	56 (75%) 14 (19%) 4 (5%) *p = 0.27*	14 (54%) 2 (8%) 6 (23%)	42 (55%) 6 (8%) 18 (24%) *p = 0.99*
**IPSS** Low Intermediate-1 Intermediate-2 High	7 (35%) 10 (50%) 2 (10%) 1 (5%)	39 (52%) 30 (40%) 5 (7%) 1 (1%) *p = 0.46*	13 (50%) 9 (35%) 3 (11%) 1 (4%)	40 (53%) 21 (28%) 14 (18%) 1 (1%) *p = 0.68*
**IPSS-R** Very Low Low Intermediate High Very High	6 (30%) 1 (5%) 6 (30%) 4 (20%) 3 (15%	19 (24%) 13 (16%) 32 (40%) 10 (12%) 5 (6%) *p* = 0.33	18 (69%) 1 (4%) 1 (4%) 0 2 (8%)	41 (54%) 4 (5%) 7 (9%) 0 (0%) 5 (7%) *p* = 0.73
**P53 R72P SNP** CC CG GG	10 (50%) 10 (50%) 0	0 39 (52%) 36 (48%)	21 (81%) 5 (19%) 0	0 34 (45%) 42 (55%)
**MDM2 SNP309** GG TT TG	13 (65%) 5 (25%) 2 (10%)	8 (11%) 33 (44%) 34 (45%)	8 (31%) 9 (35%) 9 (35%)	4 (5%) 29 (38%) 43 (57%)

*n* (%)

1 Other (RA, RARS, RCMD, RCMD-RS)

Missing data was excluded from table

### Univariate and mutlivariate analysis

We then analyzed potential variables impacting OS and PFS in non-del(5q) MDS by univariate analysis. We found that age at diagnosis, gender, and cytogenetic risk group did not predict OS. IPSS had a significant impact on survival [Hazard Ratio (HR) 2.36 (95%, CI, 1.45–3.85), *p* = 0.001], and we approached a significant impact for both WHO diagnostic category and IPSS-R [HR 1.27 (95%, CI, 0.99–1.64), *p* = 0.07; and HR 1.31 (95%, CI, 0.95–1.79), *p* = 0.09, respectively]. Furthermore, we found discrimination by the p53 functional SNP score also significantly impacted OS with an HR of 0.42 [95% CI, 0.21–0.85, *p* = 0.02]. Similar results were found for PFS in univariate analysis in which WHO diagnostic category had an HR of 1.28 [95%, CI 1.01–1.63, *p* = 0.04] while IPSS had an HR of 2.50 [95%, CI 1.56–4.00, *p* < 0.0001] and the p53 functional score had an HR 0.46 [95%, CI 0.23–0.89, *p* = 0.02] (Table 4). In our cohort, we did not reach statistical significance with IPSS-R in univariate analysis for PFS (*p* = 0.13). In the multivariate analysis, the p53 SNP functional score significantly impacted OS independent of IPSS. HR for IPSS was 2.89 [95% CI, 1.77–4.73, *p* < 0.0001] and for the p53 SNP functional score HR was 0.25 [95% CI, 0.11–0.58, *p* = 0.001] (Table 5). The significance of the p53 functional score on OS was also independent of IPSS-R. The functional score was similarly independent of IPSS for PFS, HR for IPSS was 2.84 [95% CI, 1.78–4.53, *p* < 0.0001] and for the p53 SNP functional score, HR was 0.33 [95% CI, 0.15–0.74, *p* = 0.006] (Table 5).

**Table 4:** Univariate analysis for OS and PFS

Univariate analysis for OS	HR (CI-95%)	*p*-value
Age	1.01 [0.98–1.04]	*p* = 0.62
Sex	0.89 [0.45–1.76]	*p* = 0.74
WHO classification (Others/RAEB1/RAEB2)	1.27 [0.99–1.64]	*p* = 0.07
Cytogenetic risk (Low/Int/High)	1.35 [0.77–2.36]	*p* = 0.29
IPSS (Low/Int/High)	2.36 [1.45–3.85]	*p* = 0.001
IPSS-R (VL/L/I/H/VH)	1.31 [0.95–1.79]	*p* = 0.09
TP53/MDM2 SNP Scoring System (Low/High)	0.42 [0.21–0.85]	*p* = 0.02

**Univariate analysis for PFS**	**HR (CI-95%)>**	***p*-value**
Age	1.01 [0.98–1.04]	*p* = 0.52
Sex	0.86 [0.45–1.66]	*p* = 0.66
WHO classification (Others/RAEB1/RAEB2)	1.28 [1.01–1.63]	*p* = 0.04
Cytogenetic risk (Low/Int/High)	1.49 [0.88–2.53]	*p* = 0.14
IPSS (Low/Int/High)	2.50 [1.56–4.00]	*p* < 0.0001
IPSS-R (VL/L/I/H/VH)	1.25 [0.94–1.67]	*p* = 0.13
TP53/MDM2 SNP Scoring System (Low/High)	0.46 [0.23–0.89]	*p* = 0.02

**Table 5:** Multivariate analysis for OS and PFS

Multivariate analysis for OS	HR (CI-95%)	*p*-value
IPSS (Low/Int/High)	2.89 [1.77–4.73]	*p* < 0.0001
TP53/MDM2 SNP Scoring System (Low/High)	0.25 [0.11–0.58]	*p* = 0.001

**Multivariate analysis for OS**	**HR (CI-95%)**	***p*-value**
IPSS-R (VL/L/I/H/VH)	1.29 [0.94–1.78]	*p* = 0.12
TP53/MDM2 SNP Scoring System (Low/High)	0.41 [0.20–0.84]	*p* = 0.01
**Multivariate analysis for PFS**	**HR (CI-95%)**	***p*-value**
IPSS (Low/Int/High)	2.84 [1.78–4.53]	*p* < 0.0001
TP53/MDM2 SNP Scoring System (Low/High)	0.33 [0.15–0.74]	*p* = 0.006

## DISCUSSION

In this study, we have shown that the predicted activity of p53 has prognostic importance in non-del(5q) MDS. Using a scoring system based on the predicted function of the encoded p53 protein using *TP53* R72P and *MDM2* SNP309, we found that those patients with a high p53 SNP activity score had significantly longer OS and PFS compared to those patients with a low p53 SNP functional score. In contrast, we did not find a significant impact of the p53 SNP score on outcome in patients with del(5q) MDS. However, it is possible that if those patients harboring a *TP53* mutation were excluded, the score may have greater prognostic significance. We hypothesize that *TP53* mutations will have a negative effect on our scoring system due to inefficient clearing of mutant p53 by MDM2 [25]. Unfortunately, *TP53* mutation status was not available for the patients in our data set. Analysis of this scoring system in del(5q) patients, in particular comparing *TP53* mutations vs those without, may delineate whether this scoring system should be utilized in either MDS subtype, or, should be restricted to non-del(5q) patients only. As was described by Bejar et al., [26] addition of molecular entities such as mutations should be included in current scoring systems. The authors demonstrate the negative impact of specific somatic mutations, and that presence or absence of such mutations, prognostically differentiates individuals with similar IPSS or IPSS-R scores; and, that those individuals with mutations should be placed into the next higher risk category. We similarly suggest here, that germline SNPs may also provide refined prognostication, and therefore, need to be explored further to determine whether they should also be considered. Sequencing of these SNPs by the Sanger method or by inclusion in current next-generation sequencing panels in larger validation cohorts should be used to determine whether patients harboring a low functional SNP score should similarly be upgraded to the next risk category as suggested with mutation status. These data also confirm the importance of p53 activity in these heterogeneous disorders. The association of p53 activity with respect to MDS features such as cytopenias, bone marrow blast count, cytogenetics and even clonal hematopoiesis should be explored. Potential methods to explore p53 activity include IHC or p53 nuclear localization. Although p53 IHC immunostaining has been suggested as a prognostic indicator, particularly with respect to being a surrogate marker for mutant *TP53*, [27] the utility of p53 IHC to predict p53 activity is not feasible. As p53 activity may be influenced by environmental factors, previous therapies, or previous conditions, IHC or subcellular compartmentalization at the time of diagnosis cannot provide an accurate measure of basal p53 activity. However, the presence of germline polymorphisms that can predict basal p53 functionality and that occur independent of environmental factors, may have prognostic utility.

These data underscore for the first time the importance of these SNPs in non-del(5q) MDS. Given the varied natural history of disease in MDS, identification of a genetic signature that complements IPSS to predict outcome has significant importance for treatment selection. The relationship of the *TP53* SNP score to prognostically important somatic gene mutations warrants investigation in future studies. Investigations have shown that *TP53* gene mutation is associated with lower overall response to lenalidomide and inferior overall survival in IPSS higher risk MDS patients treated with azacitidine [7, 28]. A similar analysis of our *TP53/MDM2* scoring system in relation to treatment outcome may offer further prognostic discrimination and merits investigation. Based on these data, the interaction of *TP53* R72P and *MDM2* SNP309 should be validated in a larger cohort of patients.

## MATERIALS AND METHODS

### Patients and DNA isolation

DNA was isolated from bone marrow mononuclear cells obtained from 208 MDS cases [del(5q), *n* = 102; non-del(5q), *n* = 95; unknown, *n* = 11] who provided written consent on protocols approved by the University of South Florida (USF) Institute Review Board (IRB), or equivalent, institutional approved protocols as previously described. [17] Median follow up of patients was 30 months.

### Clinical characteristics

World Health Organization (WHO) diagnostic category, and cytogenetic and International Prognostic Scoring System (IPSS) risk score was defined as previously described. [17] All cases, which included more than 90% Caucasians, were analyzed regardless of race.

### Sanger sequencing

Sequencing of *TP53* R72P was performed as previously described. [17] *MDM2* SNP309 was similarly sequenced with the primers forward 5′-CGG GAG TTC AGG GTA AAG GT-3′ and reverse 5′-AGC AAG TCG GTG CTT ACC TGG-3′ using an amplification protocol of 94°C for 2 minutes then 25 cycles at 94°C for 30 seconds, 62°C for 30 seconds and 72°C for 30 seconds, followed by 5 minutes final extension at 72°C.

### Statistical analysis

Patient characteristics and *TP53/MDM2* scoring group associations were performed using the Chi-squared test for binary variables and the Mann-Whitney test for continuous variables. OS and PFS were estimated using the Kaplan-Meier method and compared with the log-rank test. Overall survival (OS) was defined as the interval from diagnosis to the date of death. Progression-free survival (PFS) was defined as the interval from diagnosis to the date of AML progression. Multivariate analysis was performed using the Cox regression model. *P*-values below 0.05 were considered statistically significant. Statistical analyses were performed using SPSS software v22 (IBM, Armonk, NY).

## SUPPLEMENTARY TABLE


