# Supplementary material for: TP53 and MDM2 single nucleotide polymorphisms influence survival in non-del(5q) myelodysplastic syndromes
Source: Oncotarget. 2015 Sep 25;6(33):34437–45. doi: 10.18632/oncotarget.5255 (PMC4741464; doi:10.18632/oncotarget.5255)
Supplement: Supplementary file 1 [file oncotarget-06-34437-s001.pdf]

SUPPLEMENTARY TABLE

Supplementary Table S1: Patient cytogenetics

	Del(5q) MDS		Non-del(5q) MDS
001	45,XX, del(5q)	001	46,XY,del(2)(p22),inv(9)(p12q13),del(20)(q12)[4]
002	46, XX, del5(q13q33)	002	46, XY
003	del(5q), del(7q), +8	003	46, XX
004	del5(q31), +21	004	−7, 18 abn
005	del5q	005	46, XX
006	46, XX, del5(q11q33)	006	46, XY
007	del(5q), -7 , complex	007	46, XX
008	45, XX, del(5q)	008	45, X,-Y
009	46, XY, del5(q13q33)	009	46, XY
010	del(5q)	010	del(7q), t(12;17)
011	del(5q)	011	46, XY
012	46, XX, del(5q13q33)	012	46, XX
013	46, XX, del(5q13q33)	013	46, XY
014	46, XX, del(5q13q33)	014	46, XY
015	46, XY, del(5q13q33)	015	46, XX
016	del5q	016	46, XY
017	del(1p), -5, del(7q), t(8;13), -14, +mar	017	46, XY, inv (11q21;q23)
018	del5q	018	46, XX
019	44–47,XY, add(3)(p12)[15], +add(3)(p12)[4], -5[6], del(5)(q12q33)[9], add(6)(p25)[6], +8[14],+8[2], -17[15], -20[15], +22[5], +mar1[11], +mar[2] [cp15]/46,XY[5].	019	46, XY, del20(q11.2)
020	46, XX, del5(q13q35)	020	46, XY
021	46,XX, del(5)(q13q33)[4]/47, idem, +21[16]	021	46, XY
022	del(5q)	022	46, XY
023	COMPLEX	023	46, XY, +19
024	46, XX, del5(q13q33)	024	46, XY
025	44,XX,del(5)(q14q34), -7, -13, -18, +19, -20, +3mar[cp20]	025	46, XY
026	del(5q)	026	46, XY, del20(q11.2)
027	del5(q13q33), del2(p11.2p13), t(1;7)	027	46, XY
028	del5(q13q33)	028	46, XX, der22
029	-5	029	46, XY
030	unavailable	030	46, XY
031	del(5q)	031	46, XX

(*Continued*)

	Del(5q) MDS		Non-del(5q) MDS
032	unavailable	032	46, XX
033	46, XY, del5(q22q23)	033	46, XY
034	46, XX, del5(q13q33)	034	46, XY
035	del(5q)	035	46, XY
036	del(5q)	036	45, X-Y
037	46,XY,del(5)(q31q35)[2]/46,XY[cp18]	037	47, XY, +13
038	53,XY, +1, del5(q23q34), +6, +8, +9, +11, +19, +21[8]/46,XY[12]	038	46, XY
039	unavailable	039	46, XX
040	46,XX, del5(q15q33)[19]/46,XX[1]	040	46, XY
041	del(5q)	041	46, XY
042	COMPLEX	042	45, X, -Y
043	del(5q), del(20q)	043	45, XY
044	del(5q)	044	46, XY
045	43–45, XX, del(5), -7, del(10), +12, -20	045	46, XX
046	46, XY, del(5q), +17	046	46, XY
047	COMPLEX	047	46, XY, -17
048	46, XX, del5(q13q33)	048	46, XY
049	46, XX, del5(q13q33)	049	46, X, -X
050	46, XX, del5(q13q33)	050	46, XY
051	46, XX, del5(q13q33)	051	46, XY
052	46, XX, del5(q13q33), +8	052	46, XY, -20
053	46, XX, del5(q13q33)	053	46, XY
054	46, XX, del5(q13q33), +3	054	45, X, -Y
055	unavailable	055	46, XY
056	46, XX, del5(q13q33)	056	46, XX
057	46, XX, del5(q13q33)	057	45, XY, -7[20]
058	46, XX, del5(q13q33)	058	46, XY
059	46, XX, del5(q13q33)	059	46, XY, del12(p11.2)
060	46, XX, del5(q13q33)	060	46, XY
061	46, XY, del5(q13q33)	061	46, XX, t(3;9)(p22;q26)[20]
062	46, XX, del5(q13q33)	062	46, XY
063	46, XX, del5(q13q33)	063	46, XY
064	46, XX, del5(q13q33)	064	46, XY
065	46, XY, del5(q13q33)	065	46, XX
066	unavailable	066	46, XX
067	46, XY, del5(q13q33)	067	46, XX

(*Continued*)

	Del(5q) MDS		Non-del(5q) MDS
068	COMPLEX	068	46, XX, +8
069	unavailable	069	46, XX, der(12)(q13q15)
070	46, XX, del5(q13q33)	070	46, XX, der12(q13q15)
071	46,XX,del5(q13q33)[14]/ 46,XX [6]	071	46, XX
072	46,XX,del5(q31)[11]/46,XX[5]	072	46, XX, der12(q13q15)
073	46,XX,del5(q13q33)[5]/46,XX[18]	073	46, XY
074	46,XX,del5(q13q33)[3]/46,XX[5]	074	47, XY, +8
075	unavailable	075	46, XX, der12(q13q15)
076	46,XX,del5(q13q33)[6]/46,XX[14]	076	46, XY
077	46,XX, del5(q15q33)[10]; 46,XX[3]	077	46, XY
078	del(5q)	078	46, XY
079	46, XY, add1(p36), der3 t(3;5)(p12p10), -5, del9(p21), add20(q12), tmar [CP9]/46,XT	079	46, XY
080	unavailable	080	46, XX, der12(q13q15)
081	45, XX, del5(q11.2q33), add7(q36), -9, der(14;21)(q10;q10), add17(p13), 18, +21, +mar[16]/47, idem, +2mar[1]/46,XX[3]	081	46, XX, del12(q13q15)
082	44–47,XX, del5(q13q33), add7(q22), i(8)(q10), -9, add(11)(q23), -12, der(15)t(12;15)(q11;p11.2), -16, add(17)(p11.2), +mar1, +mar2[cp15]/46,XX[5]	082	46, XY, del22(q11.2q13)
083	del(5q)	083	46, XX
084	unavailable	084	46, XY
085	45, XX, del5(q15q33), del7(q22), -22[cp2]/46,XX[18]	085	inv(3q), del(20q), -Y
086	45, XX, del3(p14), -5, -7,+22[4]/46,XX[cp17]	086	46, XY
087	44, XY, del3(p13p21), del5(q12q33), del7(q22q34), -8,del(12)(p11.1),-16,-17,-18,del(20)(q11.2),+mar1,+mar2[cp21]	087	46, XY
088	unavailable	088	47, XY, +8
089	46, XX, del(5)(q15q33)[19]/46,XX[1]	089	46, X, -Y
090	unavailable	090	46, X, -Y
091	unavailable	091	46, XY
092	46, XY, del20(q11.2)[3]/46, idem, del5(q12q33)[15]/46,XY[2]	092	46, XX
093	44, XY, -3, -5, -7, +9, del11(p12)[2]/43 idem,-18,+mar[4]/46,XY[8]	093	46, XY
094	unavailable	094	44, XY, del1(p21)[2], del3(q21q26.2)[3], inv3(q21q26.2)[7], add(5)(q22), -6, add(7)(q22), -10, add(11)(q23)[2], -12, -17, +mar1, +mar2[cp12]

(*Continued*)

	Del(5q) MDS		Non-del(5q) MDS
095	43–51, X, -Y[19], add(3)(q12)[18], +del(3)(p23)[20], -5[20], -7[20], t(12;13)(p11.2;q12)[18], -21[18], -22[15], +r1[15], +mar1[14], +mar2[16], +mar3[13], +mar4[2], +1–4mar[cp20]	095	44, XY, del1(p21)[2], del3(q21q26.2)[3], inv3(q21q26.2)[7], add(5)(q22), -6, add(7)(q22), -10, add(11)(q23)[2],-12, -17, +mar1, +mar2[cp12]
096	45–46, XX, add(4)(q21), -5[3], psu dic(5;?)(q11.1;?)[16], add(8)(p23), -13[17], -16, add(17)(p11.2), -18, +r[5], +mar1, +mar2[cp20]		
097	unavailable		
098	44, XX , add(3)(q21), -5, t(7;12)(q22;p13), -8, der(16)t(8;16)(q13;q11.2)[19]/46,XX[1]		
099	unavailable		
100	unavailable		
101	unavailable		
102	unavailable		
